# Supplementary material for: Patients with affective disorders profit most from telemedical treatment: Evidence from a naturalistic patient cohort during the COVID-19 pandemic
Source: Front Psychiatry. 2022 Dec 1;13:971896. doi: 10.3389/fpsyt.2022.971896 (PMC9751940; doi:10.3389/fpsyt.2022.971896)
Supplement: Supplementary file 1 [file Data_Sheet_1.docx]

Supplementary Material

For

**Patients with affective disorders profit most from telemedical treatment: Evidence from a naturalistic patient cohort during the COVID-19 pandemic**

Tobias Rohrmann^1†^, Peter Praus^1†^, Tanja Proctor^2†^, Anastasia Benedyk^1^, Heike Tost^1^, Oliver Hennig^1^, Andreas-Meyer-Lindenberg^1^, Anna-Sophia Wahl^1,3*^

^1^Central Institute of Mental Health, University of Heidelberg, J5, 68159 Mannheim, Germany

^2^Institute of Medical Biometry (IMBI), University of Heidelberg, Im Neuenheimer Feld 130.3, 69120 Heidelberg, Germany

^3^Brain Research Institute, University of Zurich, Winterthurerstr. 190, 8057 Zurich, Switzerland

^†^shared first authorship

Corresponding author: anna-sophia.wahl@zi-mannheim.de

**Supplementary Table 1.** Table depicting psychopathological features which were reported in the medical record of the participants at the start of the telemedical treatment. Most participants revealed symptoms typical for affective disorders. N= number of subjects where information was found in the medical record. The percentage was calculated as (N/N responded)*100.

**Supplementary Table 2.** Detailed results of the two surveys of the SCL90-R before and 4-6 weeks after the beginning of the psychiatric treatment via telemedicine: The table provides the results of the three major indices of distress (GSI, PDSI and PST) as well as the subscales of the 9 psychopathological features including the number (N) of participants in the 1st and 2nd inquiry, the number of subjects with a t-values above 60 for the respective subcategory and their percentage relative to all participants ((N/N responded)*100). We also compared means between inquiry 1 and 2 for all subcategories and found a significant overall improvement (results inquiry 1 versus 2, ***P<0.001, Wilcoxon-Test was used).

### Supplementary Table 3. The table depicts the initial number of participants who gave informed consent to participate in the study and the number of subjects participating in the consecutive surveys for the WHO-5 Well Being Index, the SCL90-R and the evaluation of the telemedical treatment. The table indicates how many subjects completed all consecutive inquiries (“complete cases”) or dropped out during the course of the study (“partly complete”) or just participated in one of the inquiries (“only one inquiry completed”). The table also shows the number of participants (n=182) who gave consent to further evaluate their medical record for their psychiatric history and sociodemographics. For 174 subjects the medical records were complete and consistent in such a way that data could be further explored. All data are presented with percentage calculated relative to the initial recruited number of participants (n=254).

**Supplementary Table 1**

|  | **N** | **abnormal** |
| --- | --- | --- |
| **Signs of Psychopathology** |  |  |
| **Vigilance** | **110** | **5(4,5 %)** |
| **Orientation** | **111** | **5(4,5 %)** |
| **Memory** | **109** | **29(26,6 %)** |
| **Perception** | **108** | **5(4,6 %)** |
| **Attention** | **108** | **56(51,9%)** |
| **Concentration** | **108** | **73(67,6)** |
| **Thought process** | **106** | **76(81,7)** |
| **Thought content** | **105** | **7(6,7 %)** |
| **Tricks of the senses** | **100** | **2(2,0 %)** |
| **Self-disorder (N=132)** | **99** | **8(8,1%)** |
| **Changes in mood** | **106** | **89(84,0 %)** |
| **Ability to experience joy** | **102** | **54(52,9 %)** |
| **Lack of drive** | **102** | **58(56,9 %)** |
| **Worries, Anxiety or Fear** | **101** | **65(64,4 %)** |
| **Intrusions** | **93** | **9(9,7 %)** |
| **Compulsive behavior** | **93** | **9(9,7 %)** |
| **Psychomotor function** | **96** | **29(30,2 %)** |
| **Changes in eating habit** | **85** | **17(20,0 %)** |
| **Sleep** | **98** | **68(69,4 %)** |
| **Libido** | **70** | **29(41,4 %)** |
| **Social interaction** | **69** | **9(13,0 %)** |
| **Self-harming behavior** | **132** | **9(6,8 %)** |
| **Illness insight** | **75** | **0(0,0 %)** |

**Supplementary Table 2**

|  | **1st inquiry (N)** | **T- Value ≥ 60** | **Mean** | **1st & 2nd inquiry (N)** | **1st** | **1st Mean** | **2nd T- Value ≥ 60 (N/%)** | **2nd Mean** |
| --- | --- | --- | --- | --- | --- | --- | --- | --- |
|  |  | **(N/%)** | **(SD)** |  | **T- Value ≥ 60 (N/%)** | **(SD)** |  | **(SD)** |
| **GSI** | 234 | 180 (77,0%) | 66,61 | 83 | 64 | 66.9 | 47 | 60,42*** |
|  |  |  | -10,31 |  | -77,10% | (8.45) | -56,60% | (10.02) |
| **PST** | 233 | 151 | 62,53 | 83 | 56 | 62,02 | 42 (50,6%) | 60,18** |
|  |  | -64,80% | -9,94 |  | -67,50% | -8,48 |  | -9,25 |
| **PSDI** | 231 | 175 | 65,29 | 83 | 63 | 65,12 | 42 | 59,45*** |
|  |  | -75,70% | -8,9 |  | -75,90% | -8,23 | -50,60% | -10,67 |
|  |  |  |  |  |  |  |  |  |
| **Somatization** | 226 | 125 (55,3%) | 60,28 | 81 | 46 | 61,17 | 35 (43,2%) | 56,78*** |
|  |  |  | -10,71 |  | -56,80% | -9,33 |  | -10,08 |
| **Obsessive-Compulsive** | 225 | 184 (81,8%) | 67,55 | 81 | 69 | 68,79 | 59 (72,8%) | 64,2*** |
|  |  |  | -10,77 |  | -85,20% | -9,52 |  | -10,08 |
| **Interpersonal Sensitivity** | 224 | 151 (67,4%) | 63,67 | 81 | 55 | 63,67 | 38 (46,9%) | 59,9*** |
|  |  |  | -11,33 |  | -67,90% | -9,81 |  | -11,11 |
| **Depression** | 195 | 155 (79,5%) | 67,08 | 83 | 70 | 67,75 | 48 (57,8%) | 63,37*** |
|  |  |  | -10,61 |  | -84,30% | -9,65 |  | -10,58 |
|  |  |  |  |  |  |  |  |  |
| **Anxiety** | 226 | 160 (70,8%) | 64,35 | 79 | 54 | 64,13 | 36 (45,6%) | 58,92*** |
|  |  |  | -10,95 |  | -68,40% | -9,38 |  | -10,22 |
| **Hostility** | 224 | 144 (64,3%) | 62,46 | 81 | 36 | 62,07 | 38 (42%) | 57,47*** |
|  |  |  | -10,75 |  | -44,40% | -10,89 |  | -10,12 |
|  |  |  |  |  |  |  |  |  |
| **Phobic Anxiety** | 227 | 134 (59,0%) | 61,32 | 82 | 50 | 60,67 | 34 (41,5%) | 56,89*** |
|  |  |  | -11,56 |  | -61,00% | -9,87 |  | -11,03 |
| **Paranoid Ideation** | 224 | 125 (55,8%) | 60,33 | 80 | 39 | 60,01 | 26 (32,5%) | 54,94*** |
|  |  |  | -11,38 |  | -48,80% | -9,79 |  | -10,48 |
| **Psychoticism** | 227 | 149 (65,6%) | 62,35 | 82 | 56 | 62,55 | 36 | 57,74*** |
|  |  |  | -9,99 |  | -68,30% | -8,31 | -43,90% | -10,05 |

**Supplementary Table 3**

|  | **N or M (SD)** | **(%)** |
| --- | --- | --- |
| **Total Number (N) of recruited patients** | 254 |  |
| Female | 144 | 56,7 |
| Male | 101 | 39,8 |
| Not specified | 9 | 3,5 |
|  |  |  |
| **Participants in WHO-5 Well Being Index** |  |  |
| **Complete cases** |  |  |
| 1st inquiry | 237 | 93,3 |
| 1st and 2nd inquiry | 131 | 51,6 |
| 1st, 2nd and 3rd inquiry | 85 | 33,5 |
|  |  |  |
| **Partly complete** |  |  |
| 1st and 3rd, but not 2nd inquiry | 13 | 5,1 |
| 2nd and 3rd but not 1st inquiry | 4 | 1,6 |
|  |  |  |
| **Only one inquiry completed** |  |  |
| Only 2nd inquiry | 5 | 2 |
| Only 3rd inquiry | 1 | 0,4 |
|  |  |  |
| **Participants in SCL90-R** |  |  |
| 1st inquiry | 241 | 94,90% |
| 1st and 2nd inquiry | 83 | 32,70% |
|  |  |  |
| **Only one inquiry completed** |  |  |
| Only 2nd inquiry | 3 |  |
|  |  |  |
| **Consent received to evaluate medical records** | 182 | 71,70% |
| Evaluation of medical records completed | 174 | 68,50% |
|  |  |  |
| **Participants in evaluation survey** |  |  |
| Initial survey | 254 | 100% |
| 2nd survey | 144 | 56,7% |
| 3rd survey | 68 | 26,80% |
